# Human CRB1 and CRB2 form homo- and heteromeric protein complexes in the retina

**DOI:** 10.26508/lsa.202302440

**Published:** 2024-04-03

**Authors:** Isabel F Stehle, Joel A Imventarza, Franziska Woerz, Felix Hoffmann, Karsten Boldt, Tina Beyer, Peter MJ Quinn, Marius Ueffing

**Affiliations:** 1 Institute for Ophthalmic Research, Eberhard Karls University Tübingen , Tübingen, Germany; 2 Department of Ophthalmology, Vagelos College of Physicians & Surgeons, Columbia University; New York, NY, USA

## Abstract

*Crumbs homolog 1* (*CRB1*) is one of the key genes linked to retinitis pigmentosa and Leber congenital amaurosis, which are characterized by a high clinical heterogeneity. The Crumbs family member CRB2 has a similar protein structure to CRB1, and in zebrafish, Crb2 has been shown to interact through the extracellular domain. Here, we show that CRB1 and CRB2 co-localize in the human retina and human iPSC-derived retinal organoids. In retina-specific pull-downs, CRB1 was enriched in CRB2 samples, supporting a CRB1–CRB2 interaction. Furthermore, novel interactors of the crumbs complex were identified, representing a retina-derived protein interaction network. Using co-immunoprecipitation, we further demonstrate that human canonical CRB1 interacts with CRB1 and CRB2, but not with CRB3, which lacks an extracellular domain. Next, we explored how missense mutations in the extracellular domain affect CRB1–CRB2 interactions. We observed no or a mild loss of CRB1–CRB2 interaction, when interrogating various CRB1 or CRB2 missense mutants in vitro. Taken together, our results show a stable interaction of human canonical CRB2 and CRB1 in the retina.

## Abstract (precis)

This study describes novel interactors of the retinal Crumbs complex and reveals homo- and heterotypic interactions of CRB1 and CRB2 that are not significantly affected by patient-associated mutations.

## Introduction

Mutations in the *Crumbs homolog 1* (*CRB1*) gene cause autosomal-recessive retinitis pigmentosa (RP) and Leber congenital amaurosis, which are among the leading causes of inherited blindness (1, 2, 3, 4, 5). These retinal degenerations are characterized by a great heterogeneity regarding the onset, severity, and progression of retinal degeneration (5, 6, 7, 8, 9). No treatment is currently available to prevent or restore photoreceptor loss in these patients.

Canonical CRB1 protein structure consists of an extracellular domain with an N-terminal signal peptide, 19 EGF-like repeat domains, a single C-type lectin domain, and three laminin G domains, followed by a transmembrane and a 37-amino acid intracellular domain. The CRB1 intracellular domain comprises a FERM domain (4.1 protein, ezrin, radixin, moesin) and a C-terminal PDZ-binding motif (2, 7). Although *D. melanogaster* has only one Crumbs protein, human CRB1 is part of the CRB protein family, together with CRB2 and CRB3, which lacks the extracellular domain (9, 10). Of note, several splice forms of *CRB1* and *CRB2* have been demonstrated, yet, no specific functions have been described for individual variants in humans (6). Recently, a photoreceptor-specific isoform, CRB1-B, has been identified in the mouse and human retina that shares major parts of the extracellular domain with the canonical form CRB1-A but encodes a different intracellular domain (11). In the human retina, canonical CRB1 is localized to the outer limiting membrane (OLM) in the subapical region above the adherens junctions that connect photoreceptor cells to each other or to Müller glia cells (12). CRB1 has been detected in Müller glia cells and photoreceptor cells in the postmortem human retina, second trimester fetal human retina, and human induced pluripotent stem cell (iPSC)–derived retinal organoids (13, 14, 15). Similarly, CRB2 localizes to the subapical region of Müller glia cells and photoreceptors in the second trimester human fetal retina and human iPSC–derived retinal organoids (13, 14, 15).

The functions of canonical CRB1 have been understood mainly based on the conserved intracellular domain, which organizes a large protein scaffold (10, 12). CRB interacts with erythrocyte membrane protein band 4.1 like 5 (EPB41L5) through the intracellular FERM domain and with Lin Seven 1 (PALS1), also called membrane-associated guanylate kinase p55 subfamily member 5 (MPP5) through the PDZ domain (16, 17, 18, 19, 20). Beyond this core crumbs complex, Pals1/Mpp5 can interact with Mpp3 and Mpp4 or multiple PDZ domain protein 1 (MUPP1) and PALS1-associated tight junction, which binds to partitioning defective-6 homolog (PAR6) (16, 21, 22, 23, 24, 25). The binding of PAR6 to the PDZ domain of CRB1 leads to the recruitment of PAR3, atypical protein kinase C, and CDC42 (core PAR complex) (26, 27, 28, 29, 30, 31, 32). Through these interactions, CRB1 has been shown to be a major regulator of apical-basal polarity, OLM integrity, cell–cell adhesion, apical membrane size regulation, and cellular signaling pathways (10, 12).

Up to now, the interaction partners of the CRB1 extracellular domain have been poorly identified, and its role remains controversial. On one side, overexpression of the intracellular domain of Crumbs in *D. melanogaster* can rescue the mutant epithelial polarity phenotype to a similar extent as the full-length protein (33, 34). Likewise, the introduction of *Crb3* mRNA rescued apicobasal polarity and the retinal architecture formation in a *crb2a* mutant zebrafish (35). However, multiple studies suggest that the extracellular domain is not dispensable for CRB1 function. Zou et al showed in zebrafish that Crb2a and Crb2b preserve OLM integrity and form the cone mosaic via interaction of their extracellular domains (36). In *D. melanogaster*, the extracellular domain of crumbs was critical for stalk length formation, highlighting the importance of the extracellular domain (37, 38). Furthermore, overexpression of the transmembrane and intracellular domain of Crumbs in *D. melanogaster* removes endogenous Crumbs from the plasma membrane, suggesting a role for the extracellular domain in regulating Crumbs membrane stability (39, 40). Finally, more than 300 pathogenic or likely pathogenic variants have been described in *CRB1*, most of which are located in its extracellular domain (6, 41).

In addition to the elusive role of CRB1’s extracellular domain, the enormous clinical heterogeneity of *CRB1*-linked retinal degenerations hampers therapeutic development (42). Except for *CRB1* null variants, which are significantly associated with more severe phenotypes, and the *CRB1* c.498_506del;p.Ile167_Gly169del mutation, which is associated with the maculopathy phenotype, genotype–phenotype correlations for *CRB1* have been challenging to establish (5, 6, 7, 8, 9, 43, 44, 45, 46). In addition, Pellikka et al described variable cellular and retinal degeneration phenotypes upon introduction of missense mutations in *D. melanogaster* *Crumbs*, indicating potential allele-specific effects (47). However, as even patients with an identical *CRB1* genotype present variable disease phenotypes, the existence of additional modifier genes has been hypothesized. Except for *AIPL1*, no modifiers have been reported in humans to date (48). In the *Crb1 rd8* mouse model, *Arhgef12*, *Prkci*, *Cx3cr1*, *Mthfr*, *Jak3*, *Nfe2l2*, and *Cygb* have been described as potential modulators of disease phenotypes (49, 50, 51, 52, 53, 54). Interestingly, multiple studies highlight CRB2 as a modifier for *CRB1*-linked retinal degenerations. Illustrating this point, heterozygous loss of *Crb2* in a heterozygous *Crb1* mutant mice leads to a rather mild phenotype, whereas its loss in a homozygous *Crb1* mutant mice leads to an earlier and more severe phenotype (13). Similar results were obtained for the specific loss of *Crb2* in Müller glia cells and immature photoreceptors, which intensified the phenotype of *Crb1* mutant mice (55, 56). Recently, Boon et al showed that supplementation of human CRB1 or CRB2 by adeno-associated viral vectors was able to correct the histological and transcriptional alterations observed in patient iPSC–derived retinal organoids (57). These data emphasize the importance of CRB2 as a potential modifier regarding the development of *CRB1*-linked retinal degeneration and a potential therapeutic strategy. Nonetheless, the molecular mechanisms underlying the modulatory effect of CRB2 are currently poorly defined.

Here, we demonstrate that CRB1 and CRB2 co-localize at the OLM in the adult human retina and human iPSC–derived retinal organoids. Using co-immunoprecipitation (IP), we show homotypic and heterotypic interactions of human canonical CRB1 and CRB2 but not CRB3, indicating that the CRB extracellular domain is essential for this interaction. By exposing FLAG-tagged CRB2 to porcine retinal lysate followed by mass spectrometry, we confirm the pull-down of the porcine CRB1 complex and previously undescribed interactors involved in vesicular transport, signaling, lipid metabolism, cilia homeostasis, and cytoskeleton. Furthermore, we show that *CRB1* and *CRB2* patient missense mutations located in the extracellular domain do not significantly impair this interaction in vitro, suggesting a strong CRB1–CRB2 interaction.

## Results

### CRB1 and CRB2 co-localize at the OLM in the adult human retina and human iPSC–derived retinal organoids

**Figure 1.**
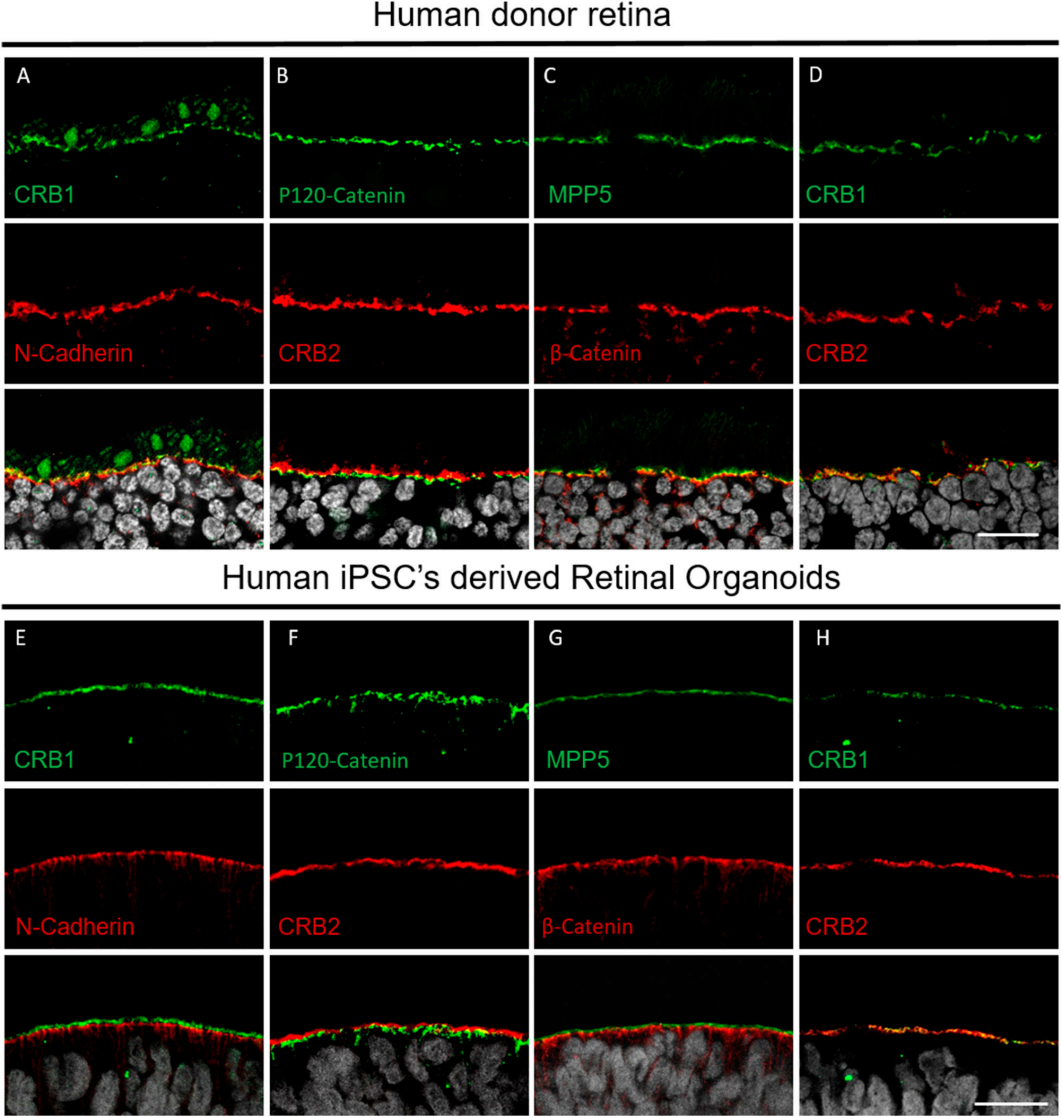
Localization of the CRB complex at the outer limiting membrane in adult human retina and human iPSC–derived retinal organoids. **(A, B, C, D, E, F, G, H)** Immunohistochemistry images of adult human retina (A, B, C, D) and hiPSC-derived retinal organoids (E, F, G, H). **(A, B, C, D, E, F, G, H)** Sections were stained for subapical region markers CRB1 (A, D, E, H), CRB2 (B, D, F, H), and MPP5 (C, G) and adherens junction markers N-cadherin (A, E), ZO-1 (B, F), and β-catenin (C, G). OLM, outer limiting membrane. Scale bars, 20 μm.

To confirm previous findings on the localization of CRB complex members at the OLM of the retina (13, 14), we undertook immunohistochemistry studies in the adult human donor retina and differentiation day 200 (DD200) human iPSC (hiPSC)–derived retinal organoids (Fig 1). In the adult human retina, the CRB complex members CRB1, CRB2, and MPP5 localized to the subapical region adjacent to adherens junction markers N-cadherin, zonula occludens-1 (ZO-1), and β-catenin, respectively (Fig 1A–C). Furthermore, we found that CRB1 and CRB2 co-localized together at the subapical region in the adult human retina (Fig 1D). The protein localization pattern for CRB complex members CRB1, CRB2, and MPP5 and adherens junction markers N-cadherin, ZO-1, and β-catenin was recapitulated in DD200 hiPSC-derived retinal organoids (Fig 1E–H). Taken together, those data highlight the crucial role that both CRB1 and CRB2 have at the OLM of the human retina.

### Human CRB1 binds to the Crumbs family member CRB2 but not CRB3

**Figure 2.**
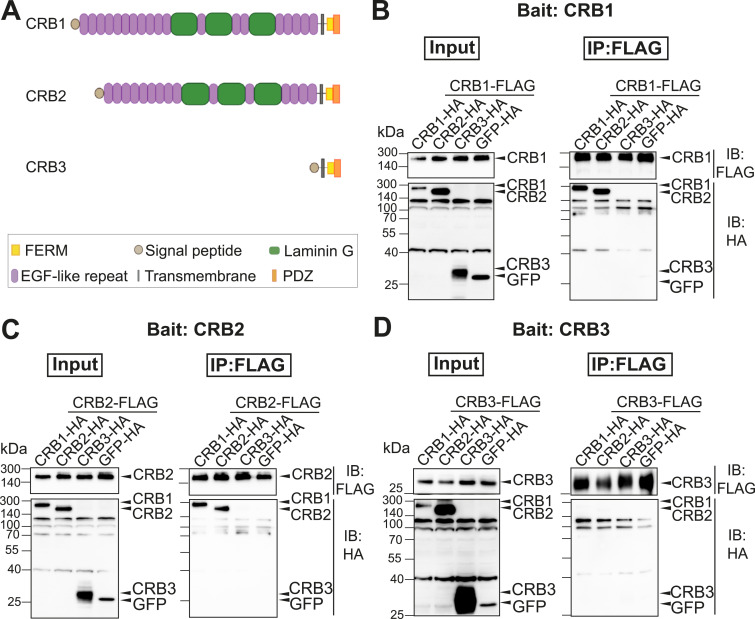
CRB1 and CRB2, but not CRB3, interact homo- and heterotopically. **(A)** Schematic overview of the Crumbs protein family members canonical CRB1, CRB2, and CRB3. Colors represent the predicted protein domain. **(B, C, D)** CRB1-FLAG (B), CRB2-FLAG (C), CRB3-FLAG (D) were co-transfected with CRB1-HA, CRB2-HA, CRB3-HA, or GFP-HA as the negative control in HEK293T cells. Inputs (left panel) and eluates after FLAG-IP (right panel) were analyzed by Western blot analysis. Representative blots of three independent experiments are depicted. IB, immunoblot; IP, immunoprecipitation.

Given the specific localization of CRB1 and CRB2 above the OLM, we next assessed whether CRB1 interacts with CRB2 and CRB3. CRB1 and CRB2 both have a large extracellular domain that is absent in CRB3 (Fig 2A). To study the interaction between canonical CRB1, CRB2, and CRB3, co-immunoprecipitation (IP) assays were performed by pairwise overexpression of those proteins fused to FLAG or HA tags in HEK293T cells. Western blot analysis showed that pulled-down CRB1-FLAG associates with CRB1 and CRB2 but not with CRB3 (Fig 2B). Similarly, we observed an interaction of CRB2 with CRB1 and CRB2 but not CRB3 when CRB2 was used as bait (Fig 2C). No interaction was shown for CRB3 with CRB1, CRB2, or CRB3 (Fig 2D). Together, these data suggest homotypic and heterotypic interaction of canonical CRB1 and CRB2 but not CRB3, suggesting that the extracellular domain is required for this interaction.

### CRB2 interacts with endogenous CRB1 in retinal tissue

**Figure 3.**
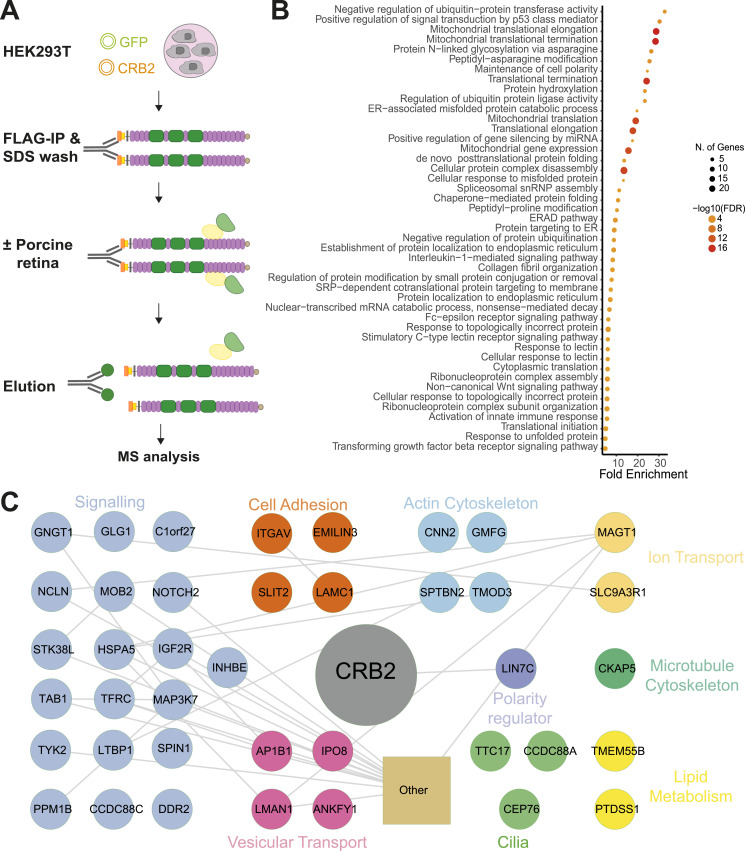
Protein–protein interaction network of CRB2 after stringent SDS washing but without incubation of porcine retina. **(A)** CRB2-FLAG or GFP-FLAG were transiently overexpressed in HEK293T cells followed by FLAG-IP. Bait proteins were washed and purified with 0.01% SDS followed by incubation with or without porcine retinal lysate. Upon elution, samples were analyzed by mass spectrometry. **(B)** The top 50 significant pathways (FDR < 0.05) identified using gene ontology biological process overrepresentation analysis with CRB2 interaction partners without porcine retinal lysate incubation generated with ShinyGO (one-sample *t* test, FDR 0.05, log_2_ ratio [CRB2/GFP]>2) (60). **(C)** String and Cytoscape analysis of significant CRB2 interactors from HEK293T cells remaining after SDS washing (61, 62). Colors represent biological processes based on Uniprot database and the literature (63). Grey lines show reported interactions based on the database/experiment. Proteins involved in mitochondria, transcription, DNA binding, splicing, and chaperones are summarized. All interactors are shown in Table S1 N = 6.

Our findings on the interaction between CRB1 and CRB2 provide a first hint on a possible interdependence of CRB2 and canonical CRB1. To further investigate the role of the CRB1–CRB2 interaction in the retina, we next investigated the retinal protein–protein interaction network of CRB2. To this end, we transiently overexpressed human CRB2-FLAG or GFP-FLAG (control) in HEK293T cells. After FLAG-IP, bait proteins immobilized on beads through an anti-FLAG antibody were washed, purified with 0.01% SDS, and incubated with or without porcine neural retina lysates, as previously described (58, 59). Protein complexes were then eluted from the beads and analyzed by mass spectrometry (Fig 3A).

**Figure S1.**
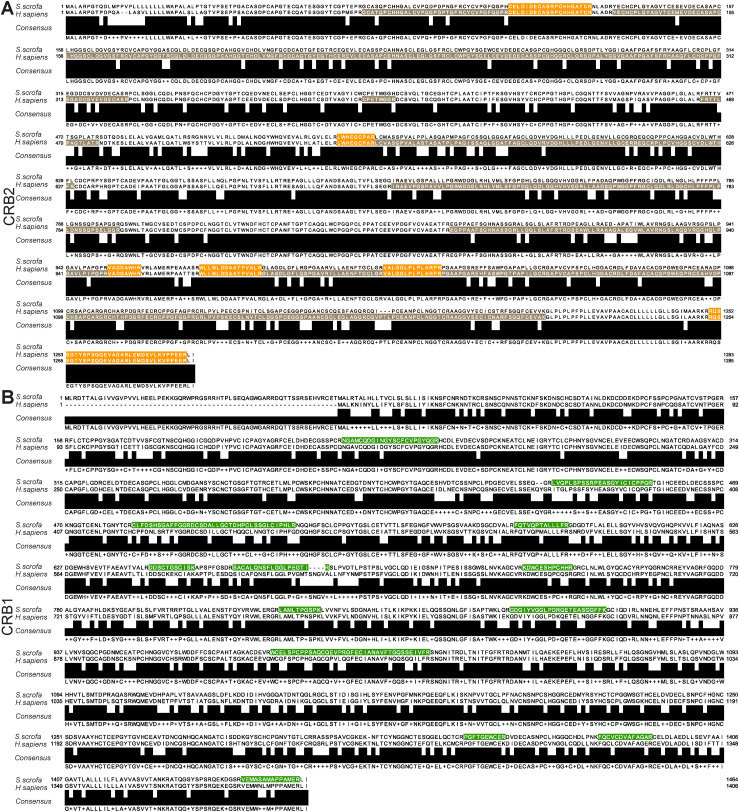
Specific peptides detected for CRB1 and CRB2 by mass spectrometry. **(A)** Alignment of CRB2 protein sequence from *Homo sapiens* (*H. sapiens*, bottom sequence, NP_775960.4) and *Sus scrofa* (*S. scrofa*, upper sequence, XP_003122203.3) together with the consensus sequence. Peptides identified by mass spectrometry are highlighted in brown (specific to *H. sapiens*) and orange (shared *H. sapiens* and *S. scrofa*). **(B)** Alignment of CRB1 protein sequence of *H. sapiens* (bottom strand) and *S. scrofa* (top strand). Specific peptides identified are highlighted in green.

We first examined CRB2 and its associated protein abundance after stringent SDS washing but without the addition of porcine retina. This allowed to identify interactors that were derived from HEK293T cells for subsequent comparison with retina-derived interactors. CRB2 bait was detected by mass spectrometry with a peptide coverage of at least 42% (Fig S1A). In addition, 146 proteins derived from HEK293T cells remained significantly enriched in CRB2 samples compared with the control, indicating a strong interaction with CRB2 (Table S1). Gene Ontology (GO) biological process analysis revealed that these interactors were enriched as being involved in cell polarity (LIN7C), translation termination, and protein targeting to the ER (Fig 3B). In addition, processes most likely linked to transient overexpression including cellular response to misfolded protein and ERAD pathway were significantly enriched. We also detected a high abundance of proteins linked to signal transduction through p53, interleukin-1–mediated signaling, C-lectin receptor signaling, Notch signaling, non-canonical WNT signaling, and TGF-β receptor signaling (Fig 3B and C). Furthermore, proteins involved in cell adhesion, actin and microtubule cytoskeleton, ion transport, cilia, vesicular transport, and lipid metabolism were identified as highly abundant in CRB2 samples after SDS washing from HEK293T cells (Fig 3B and C).

Table S1 List of proteins identified by mass spectrometry in CRB2 bait samples compared to control without porcine retinal lysate incubation.

**Figure 4.**
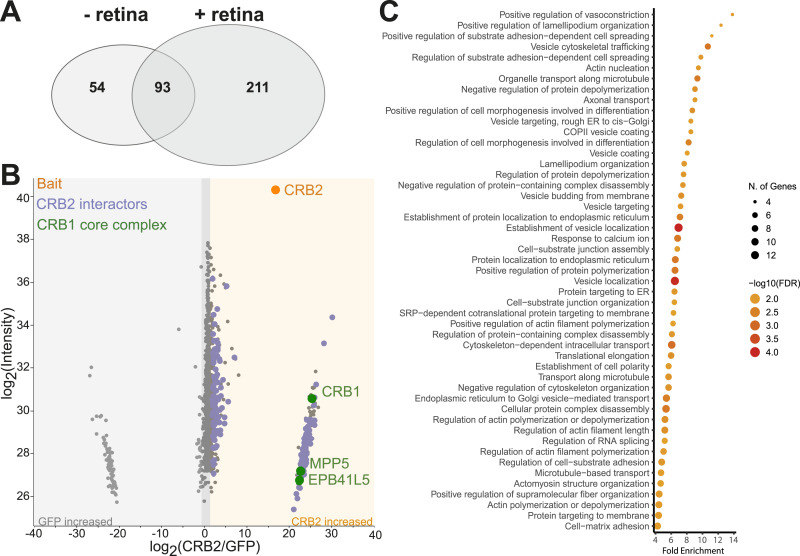
Retinal protein–protein interaction network of CRB2 includes the CRB1 core complex. **(A)** Venn diagram of proteins with significant CRB2 interacting proteins with and without incubation with the porcine retina (one-sample *t* test, FDR 0.05, log_2_[CRB2/GFP]>2). **(B)** Scatter plot of log_2_ ratios of proteins identified in CRB2-FLAG bait samples against control upon incubation with porcine retina lysate. The bait protein CRB2 is depicted in orange. Proteins with significantly higher abundance in CRB2 bait samples are shown in purple (one-sample *t* test, FDR 0.05, log_2_ ratio [CRB2/GFP]>2). CRB1 and core crumbs complex members identified as significantly more abundant are depicted in green. **(C)** The top 50 of the significant pathways (FDR < 0.05) identified using gene ontology biological process overrepresentation analysis with CRB2 interaction partners generated with ShinyGO (60). N = 6.

Next, we explored the retina-specific protein–protein interaction network of human CRB2 upon incubation with porcine neural retina lysate. Human and porcine CRB2 share 84.5% of their amino acid sequence, indicating a high degree of conservation between the two species (Fig S1B). Furthermore, we detected 304 proteins with a significantly higher abundance in CRB2 samples compared with the control after incubation with the porcine retina (one-sample *t* test, FDR 0.05, log_2_ ratio [CRB2/GFP]>2) (Fig 4A and Table S2). Of these, 211 proteins were specifically detected when CRB2 was incubated with porcine retina, whereas 93 proteins were also identified without incubation of the porcine retina and were therefore considered to be HEK293T cell interactors (Fig 4A). Of the 211 retinal CRB2 interactors, we observed a significantly higher abundance of CRB1 and the core crumbs complex members MPP5 and EPB41L5 in CRB2 samples compared with the control (Fig 4B). As CRB1 is not expressed in HEK293T cells according to the Human Protein Atlas, we expected CRB1 to be porcine retina–derived (64). To further verify this, we analyzed the specific peptides detected for CRB1. Although porcine CRB1 and human CRB1 are 74.3% identical in amino acid sequence, the peptides detected for CRB1 confirmed the interaction of porcine CRB1 with human CRB2 (Fig S1B). The above data indicate a strong interaction of CRB1 and CRB2 in a retinal context.

Table S2 List of proteins identified by mass spectrometry in CRB2 bait samples compared to control upon porcine retinal lysate incubation.

### Protein–protein interaction analysis identifies novel retinal complex members of the human crumbs complex

**Figure 5.**
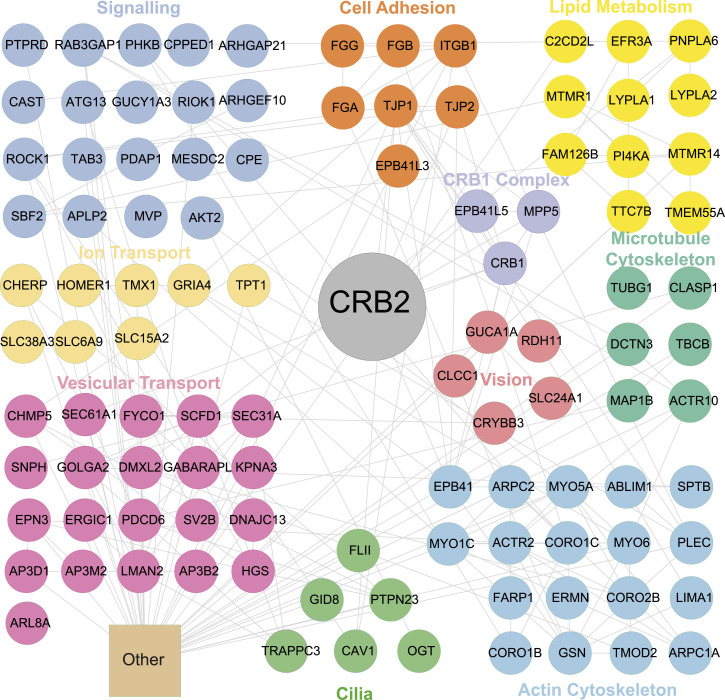
CRB2 protein–protein retinal interaction network includes proteins involved in signaling, lipid metabolism, cytoskeleton, cilia, and ion transport. A significantly higher abundance of proteins in CRB2 bait samples compared with GFP control exposed to porcine retina lysate was found and analyzed by String and Cytoscape (61, 62). Colors represent biological processes based on Uniprot database and the literature (63). Grey lines show reported interactions based on the database/experiment. Proteins involved in mitochondria, transcription, DNA binding, splicing, and chaperones are summarized as other. All interactors are shown in Table S2.

To gain further insights into the potential functions of the Crumbs complex in the retina, we further explored the 211 proteins that specifically interact with CRB2 upon incubation with porcine retina. Besides the CRB1 core complex members, GO biological process enrichment further showed a strong enrichment of proteins involved in cell–cell or cell–matrix adhesion processes, cell polarity, actin and microtubule cytoskeleton, vesicular transport, and targeting (Figs 4C and 5). Furthermore, proteins with a described function in ion transport, lipid metabolism including phosphoinositide metabolism, signaling, vision, and cilia-associated proteins were identified with significantly higher abundance in CRB2 than in the control (Fig 5). Collectively, these data provide novel candidate members of the retinal crumbs complex and suggest potential functions in the retina.

### *CRB1* and *CRB2* patient-associated missense mutations only mildly affect CRB1–CRB2 interaction in vitro

**Figure 6.**
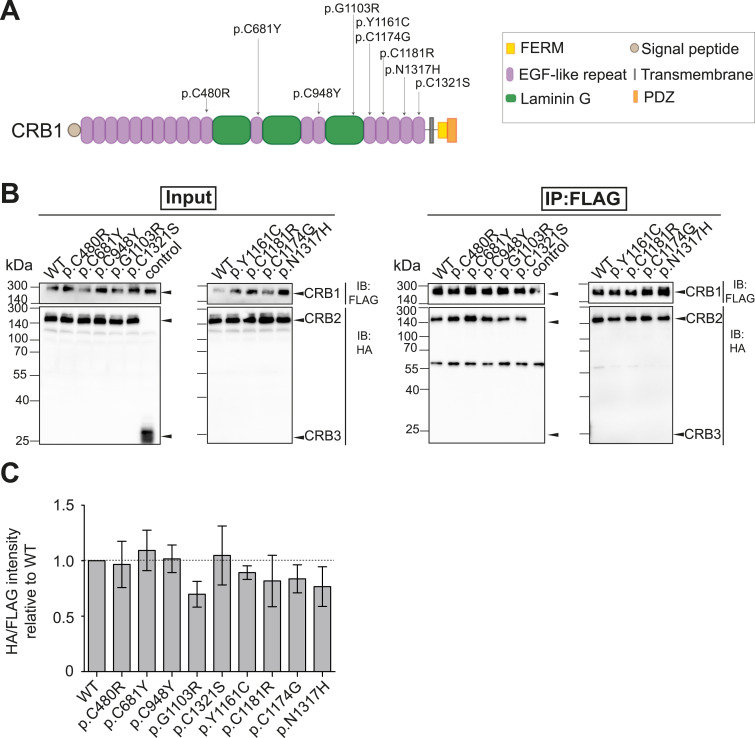
Most of the CRB1 missense mutations tested mildly alter CRB1–CRB2 interaction. **(A)** Graphical representation of the missense mutation introduced in the CRB1 construct. **(B)** CRB1-FLAG constructs WT or mutant were co-transfected with CRB2-HA in HEK293T cells. CRB3-HA was co-transfected as the control. Upon FLAG-IP, input (Left panel) and eluates (right panel) were analyzed by Western blot with HA and FLAG antibodies. **(C)** Quantification of the signal intensity HA/FLAG in eluates upon FLAG-IP relative to WT of three biological replicates. Data depict mean ± SEM.

Most of *CRB1*-associated mutations in patients with autosomal-recessive inherited retinal degenerations affect the CRB1 extracellular domain. As the data presented above indicate a role of the extracellular domain in the CRB1–CRB2 interaction, we next assessed whether patient-associated *CRB1* missense mutations alter the CRB1–CRB2 interaction. Therefore, we introduced various *CRB1*-specific patient missense mutations (p.C480R, p.C681Y. p.C948Y, p.G1103R, p.Y1161C, p.C1174G, p.N1317H) by site-directed mutagenesis into the canonical CRB1 construct (Fig 6A). Mutations were selected to cover the region of the extracellular domain that is conserved in the three major CRB1 isoforms expressed in the retina as described by Ray et al (11). Next, the CRB1-FLAG WT or mutant (MT) and CRB2-HA WT constructs were co-transfected in HEK293T cells followed by FLAG-IP and Western blot analysis.

Overall, none of the mutations showed a significant loss or gain of CRB1–CRB2 interaction in vitro (Fig 6B and C). We observe that CRB1 p.C681Y, p.C984Y, and p.C1321S show similar binding capacity to CRB2 compared with CRB1 WT. Similarly, CRB1 p.C480R retains 96% of the interaction capacity with CRB2 relative to CRB1 WT. A mild reduction of 11%, 19%, 17%, and 24% was observed for CRB1 p.Y1161C, p.C1181R, p.C1174G, and p.C1317H, respectively. CRB1 p.G1103R, which is located in the last laminin G domain, impairs CRB1–CRB2 binding by ∼30% compared with CRB1 WT.

**Figure S2.**
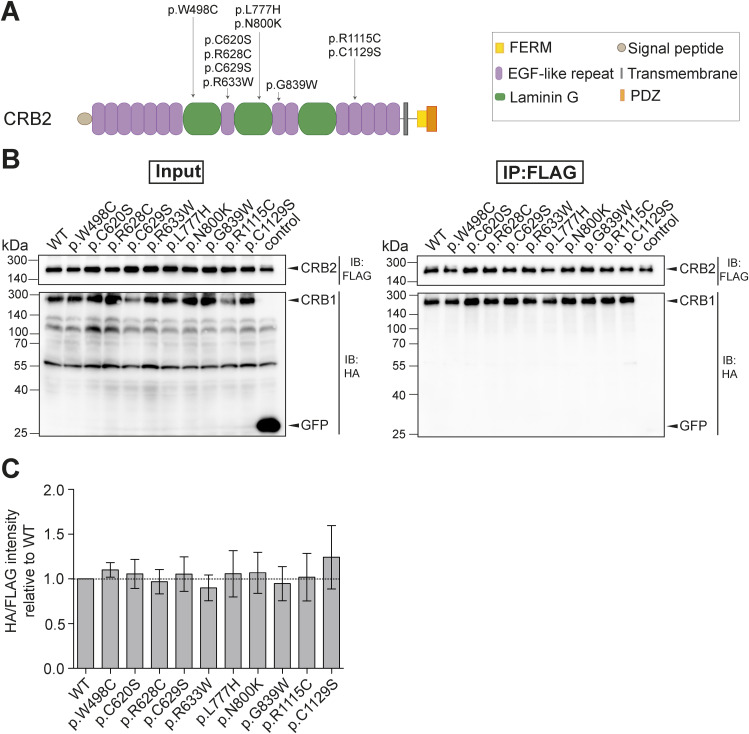
Patient-associated missense mutation in CRB2 do not significantly affect CRB2-CRB1 interaction. **(A)** Presentation of the CRB2 protein structure and the patient-associated missense mutations that were introduced by SDM. **(B, C)** CRB2-FLAG MT were co-transfected with CRB1-HA WT in HEK293T cells. Lysates (Input, left panel) and eluates after FLAG-IP (right panel) were analyzed by Western blot. IB, immunoblot; IP, immunoprecipitation. **(C)** Quantification of the intensity of HA/FLAG signals in eluates of three biological replicates. Mean ± SEM are shown.

Because most of the *CRB2* mutations described in patients with *CRB2*-related syndromes are likewise located in the extracellular domain, we next examined the effect of *CRB2* missense mutations on the CRB1-CRB2 interaction (Fig S2A) (65). To this end, a co-IP experiment was performed in which CRB2-FLAG WT or MT was co-transfected with CRB1-HA WT in HEK293T cells. After FLAG-IP, we observed no significant influence of *CRB2* missense mutations on the interaction of CRB2 and CRB1 (Fig S2B and C).

Taken together, these data provide evidence that most of the patient missense mutations in *CRB1* or *CRB2* analyzed do not or only mildly affect CRB1–CRB2 interaction in vitro, indicating a strong CRB1–CRB2 interaction.

## Discussion

In this study, we investigated the interaction of canonical CRB1 and CRB2 in the retina. First, we confirmed that canonical CRB1 and CRB2 co-localize at the OLM in human iPSC–derived retinal organoids and adult human retina. Second, we showed by co-IP that canonical CRB1 and CRB2 interact homo- and heterotypically but not with CRB3, indicating the necessity of the extracellular domain for this interaction. Third, the CRB2 retinal protein–protein interaction network included CRB1 and the core CRB complex members MPP5 and EPB41L5, corroborating this interaction in a retinal context. The identified retinal protein interactions linked the crumbs complex to signaling, ciliary homeostasis, actin and microtubule cytoskeleton, lipid metabolism, cell adhesion, and ion transport. The presence of interactions in the retina may be the basis for a unique, organ- and tissue-specific function of CRB1 and CRB2. Fourth, various patient-described missense mutations in *CRB1* or *CRB2* did not or only mildly altered the CRB1–CRB2 interaction in vitro, hinting toward a strong CRB1–CRB2 interaction.

To date, 37 cases with *CRB2* mutations have been described (65, 66, 67, 68, 69, 70, 71, 72, 73). Similar to *CRB1* mutations, most of these mutations are located in the extracellular domain (65). Given the expression of CRB2 in multiple epithelial-derived tissues, patients present with phenotypes ranging from severe prenatal abnormalities to postnatal isolated renal abnormalities with few cases of retinal involvement (70). *CRB2*-linked pathology has been debated as a ciliopathy-like syndrome as the pathological defects are comparable to those described in ciliopathy patients (70). CRB2 has been among others detected in vesicles near the centrosome in ARPE19 cells and has been shown to be required for primary ciliary vesicle formation and anchoring (74 *Preprint*). In zebrafish, Crb2 is required for the elongation of the photoreceptor inner segment and of the primary cilium of kidney cells (35, 75). Similarly, CRB3 was shown to regulate ciliogenesis and cilium-related signaling in cell lines including MDCK cells, MCF10A, and mammary and kidney tissues in a *Crb3* KO mouse model (76, 77, 78). In the human retina, CRB2 is localized presumably in the striated ciliary rootlets at the apical tips, and CRB3A was found in the ellipsoid region near the basal body (79). The protein–protein interaction network of CRB2 described here contained proteins with previously described roles in ciliogenesis, such as CCDC88A/Girdin, GID8, and PTPN23, supporting a cilia-related function of CRB2 (80, 81, 82).

Besides its interaction with ciliary proteins, we detected CRB1 and the Crumbs complex proteins as part of the CRB2 protein interaction network. In addition, we showed that canonical CRB1 and CRB2 co-localized at the OLM in the human retina, consistent with previous findings by Quinn et al (14). We demonstrated that human canonical CRB1 and CRB2 not only co-localize but also interact homo- and heterotypically, suggesting synergistic functions of the heterotypic Crumbs complex in the retina. Because CRB3, which lacks the extracellular domain, did not interact with CRB1 and CRB2, the extracellular domain might be crucial for this interaction. Our data are consistent with observations by Zou et al in zebrafish, where Crb2a and Crb2b adhere through the extracellular domain (36). Future experiments will need to determine whether CRB proteins form dimeric or multimeric complexes and whether these interactions happen *cis*- or *trans* or both. This will also provide further insights into the function of the CRB–CRB interaction. One could hypothesize that homo- and heterotypic CRB1–CRB2 interactions function as (1) regulators of CRB membrane stability and localization, (2) transmembrane receptors that serve as signaling modulators between different retinal cell types, or (3) cell–cell adhesion molecules between photoreceptors and Müller glia cells, thereby contributing to the OLM stability and cell–cell communication.

To date, it has been hypothesized that the extracellular domain is involved in the membrane stability of Crumbs. In *D. melanogaster* germline epithelium, overexpression of the extracellular domain to GFP localizes normally, whereas overexpression of the intracellular domain causes most of the endogenous Crumbs to be endocytosed (39). In addition, two recent publications show that missense mutations in the extracellular domain of *CRB1* lead to a reduction of CRB1 at the OLM in patient iPSC–derived organoids, hinting toward a trafficking deficit to the OLM or increased turnover (57, 83). These data led to the hypothesis that missense mutations in the CRB1 extracellular domain could impair the CRB1–CRB1 or CRB1–CRB2 interaction required for stable localization to the OLM. Although this remains a possibility, we have shown here that a panel of CRB1 missense mutations localized in the extracellular domain, including C948Y described by Boon et al, had no or only a minor effect on CRB2 binding in vitro. Similarly, missense mutations described in the extracellular domain of *CRB2* did not significantly abolish the CRB1–CRB2 interaction. Since we and others have also shown that the extracellular domain is important for CRB–CRB interaction, we propose that multiple amino acids or protein domains in the extracellular domain contribute to stable CRB1–CRB2 binding. Furthermore, variants that completely disrupt the interaction, in particularly of CRB2, may be lethal. To illustrate this point, complete KO of *Crb2* in mice is embryonic lethal and loss of *Crb2* impairs the survival and differentiation of ESC-derived neuronal progenitors (84, 85). Finally, it now remains crucial to assess the influence of those missense mutations under physiological condition or photoreceptor cell stress. A mild reduction in vitro might have a significant impact at the OLM depending on the turnover of CRB1 at the apical side.

As CRB3, which lacks the extracellular domain, is able to localize to the membrane, it is reasonable to infer additional functions of the extracellular domain beyond regulation of membrane stability. For various transmembrane receptors, dimerization can be either promoted after ligand binding or required before ligand binding to induce downstream signaling activity (86). Recently, an interaction between the extracellular domains of CRB1 and NOTCH1 was found at the OLM in control iPSC–derived retinal organoids, and NOTCH1 levels were shown to be reduced at the OLM similar to CRB1 in *CRB1* patient iPSC–derived retinal organoids but increased in the ONL (83). In concurrence with these findings, studies in zebrafish and *D. melanogaster* have shown that the extracellular domain of CRB binds to the extracellular domain of Notch and prevents Notch endocytosis (87, 88, 89). In line with those findings, we have identified NOTCH2 as a human CRB2 interactor in HEK293T cells. NOTCH2 is one of the 54 proteins that were specifically identified as a CRB2 interactor without incubation of porcine retinal lysate. A likely possibility is that the healthy adult porcine retina only expresses low levels of NOTCH receptors as they are highly expressed during development and in Müller glia cells upon injury (90). The TopTen LC-MS/MS data-dependent acquisition used in this study is biased toward identifying the most abundant proteins, and thus, these low-abundant interactors may not be detected. In addition, dynamic protein interactors from HEK293T cells may be replaced by porcine retinal interactors that have a higher affinity for CRB2. This could be further investigated using the data-independent analysis method, targeted mass spectrometry, or Western blot analysis if working antibodies are available. For NOTCH2, it would be interesting to further address whether NOTCH interacts with CRB as a monomer, dimer, or multimer.

Third, the interaction of CRB1–CRB2 through the extracellular domain provides evidence that human CRB proteins function as a new class of cell–cell adhesion molecules similar to the cadherin protein family, as suggested for zebrafish by Zou et al (36). In agreement with this, expression of zebrafish CRB2a and CRB2b was shown to induce cell aggregation through their extracellular domains in HEK293T cells (36). Various studies have also shown that CRB1 is crucial for the development and integrity of the OLM in *D. melanogaster* and mice (17, 37, 91, 92, 93). Similarly, patient iPSC–derived retinal organoids at certain age display ectopic photoreceptor localization in regions of OLM disruption (14, 57). CRB1 and CRB2 homo- and heteromerization may therefore directly contribute to the establishment and maintenance of the OLM.

The protein–protein interaction network described here provides new evidences into the functions executed by the crumbs complex in the retina. We have identified multiple proteins involved in phosphoinositide (PI) metabolism as part of the CRB retinal protein–protein interaction network. Various studies have shown that dysregulation of PIs leads to blindness, emphasizing their importance in the retina (94). Lattner et al previously showed that loss of CRB leads to accumulation of PI(4,5)P_2_ in *D. melanogaster* salivary gland epithelia, which impairs the apical secretion and trafficking function (95). Lattner et al demonstrate that loss of PTEN, which converts PI(3,4,5)P_3_ to PI(4,5)P_2_, prevents accumulation of PI(4,5)P_2_ in CRB1 knockdown salivary glands and hypothesize indirect regulation of PTEN by the CRB interactors MyoV and β-Spectrin (95). The exact molecular mechanisms remain unknown. We and others have not detected an interaction between Pten and CRB (95). However, we identified TMEM55A and B (also known as PIP4P2 and PIP4P1), which are involved in the conversion of PI(4,5)P_2_ to PI(5)P, as interactors of CRB in HEK293T and upon incubation with porcine retinal lysate. As reduction of TMEM55 activity can also lead to the accumulation of PI(4,5)P_2_, it would be of high interest to assess how CRB regulates TMEM55 and whether targeting of TMEM55 could restore phenotypes in *D. melanogaster* salivary gland epithelia and potential retinal phenotypes.

Besides signaling, PI plays an important role in regulating vesicular trafficking as part of the endosomal pathway (96, 97). In *D. melanogaster*, alterations in the endolysosomal system organization were found to precede light-induced degeneration (98). Recently, Buck and colleagues found that CRB1 plays a role in early endosome maturation receptor recycling in the retina (83). We have detected multiple proteins involved in vesicular trafficking, for instance, ARL8A, as part of the CRB2 protein–protein interaction network. ARL8A regulates lysosome motility, and Arl8+ vesicles have been shown to accumulate in CRB1 patient-derived retinal organoids and in the *D. melanogaster* CRB model before light-induced degeneration (57, 83, 98, 99). Furthermore, Boon et al demonstrate alterations in the endosomal pathway in CRB1 patient-derived retinal organoids and CRB1 and CRB2 KO retinal organoids using single-cell RNAseq, which can be rescued by AAV-mediated CRB2 or CRB1 gene delivery (57, 100).

In conclusion, in this study, we confirm the human canonical CRB1 and CRB2 homo- and heteromerization in the retina and provide novel candidate interactors of the retinal crumbs complex.

## Materials and Methods

### Cell lines and culture

HEK293T cells (CRL-3216; American Type Culture Collection) were cultured in DMEM (Sigma-Aldrich) supplemented with 10% FBS (Sigma-Aldrich) and 0.5% penicillin/streptomycin (Gibco). Cells were cultured at 37°C in a 5% CO_2_ incubator and analyzed for *Mycoplasma* by PCR.

### Human iPSC–derived retinal organoid differentiation

Previously validated and published WT hiPSC line (101) was maintained on Matrigel (BD)-coated plates in mTeSR Plus medium (STEMCELL Technologies) and passaged with ReleSR (STEMCELL Technologies). The retinal organoid differentiation was carried out using the agarose microwell array seeding and scraping (AMASS) method with previously described minor modifications (101, 102). In brief, iPSC at 90% confluence were detached with ReleSR (STEMCELL Technologies). Cells were counted and seeded at 2,000 cells per microwell and incubated with (±) blebbistatin in mTeSR Plus medium overnight. Over the next three differentiation days (DD), the medium was transitioned from mTeSR plus to Neural Induction Medium 1 (NIM)-1, forming embryoid bodies (EBs). On DD7, EBs were moved to Matrigel-coated wells until DD28. On DD16, the medium was transitioned from NIM-1 to NIM-2. The checkerboard-scrapping method was used to lift the neuroepithelia. The lifted retinal organoids were maintained until DD41 in NIM-2 on poly-HEMA (Sigma-Aldrich)–coated wells and changed to retinal lamination medium 1 (RLM-1) from DD42 to DD69. RLM-2 was used from DD70 to DD97 and RLM-3 was from DD98 for long-term culture. NIM1 (50 ml): 48.95 ml DMEM/F12, 10 μl 10 mg/ml heparin (final concentration, 2 μg/ml), 0.5 ml Media-Non-Essential Amino Acids (100×, MEM NEAA), and 0.5 ml N2 supplement (100×). NIM2 (50 ml): 48 ml DMEM/F12 (3:1), 0.5 ml MEM NEAA, 1 ml B27 Supplement (50×, minus vitamin A), and 0.5 ml penicillin-streptomycin (P/S, 10,000 U/ml). RLM1 (50 ml): 42.9 ml DMEM/F12 (3:1), 0.1 ml taurine (100 μM final concentration), 5 ml FBS, 1 ml B27, 0.5 ml MEM NEAA, and 0.5 ml P/S. RLM2: RLM1 supplemented with 0.1 μl per mL of 10 mM retinoic acid. RLM3: RLM1 without B27, replaced with N2 supplement, and retinoic acid reduced to 0.05 μl per ml.

### Immunofluorescence

The human donor retina was isolated from human autopsy eye shells purchased from the Eye-Bank for Sight Restoration. The donor retina was fixed with 4% PFA for 30 min and then cryoprotected with 15% and then 30% sucrose. Subsequently, the donor retina was embedded in Tissue-Tek O.C.T. Compound (Sakura, Finetek) and 10-μm cryosections were performed. DD 200 iPSC-derived retinal organoids were washed twice in PBS before being paraffin-embedded, and 5-μm paraffin sections made. After 24-h air dry, paraffin sections were baked for 2 h in a dry oven at 60°C. In a fume hood, sections were deparaffinized with three fresh xylene baths for 5 min and occasional agitation. Slides were then incubated for 5 min in two 100% alcohol baths and transferred to distilled water to be rehydrated. Antigen retrieval was performed in a steamer for 20 min with antigen unmasking solution, citrate-based (H-3300, 1:100; Vector Laboratories). Immediately after, slides were transferred to a room temperature distilled water bath. Both cryosections and paraffin slides were rehydrated in PBS and blocked for 1 h with a solution containing 10% normal goat serum, 1% BSA, and 0.4% Triton X-100 in PBS. The following antibodies were used: CRB1 (#23418-A01; Abnova), CRB2 (#PA5-25628; Invitrogen), ZO-1 (#33-9100; Invitrogen), N-cadherin (#AB18203; Abcam), β-catenin (#C610153; BD Transduction), and MPP5 (#177-10-1-AP; ProteinTech). Antibodies were diluted in 0.3% normal goat serum, 0.4% Triton X-100, and 1% BSA in PBS and incubated overnight at 4°C. After washing with PBS, sections were incubated for 1 h at room temperature with fluorescent-labeled goat anti-mouse Alexa Flour 488 and goat anti-rabbit Alexa Flour 555 secondary antibodies (cryosection: 1:1,000, paraffin sections: 1:400) diluted in 1% BSA in PBS. Cryosection samples were counterstained and mounted using VECTASHIELD Vibrance Antifade Mounting Medium with DAPI (H-1800; Vector Laboratories). The paraffin slides were counterstained with DAPI for 5 min at room temperature (1 mg/ml; Thermo Fisher Scientific). After washing with PBS, slides were mounted using VECTASHIELD Vibrance Antifade Mounting Medium (H-1700; Vector Laboratories). Images were taken using a confocal microscope (LSM800 microscope; Zeiss).

### DNA constructs

Human *CRB1* (NM_202153), *CRB2* (NM_173689), and *CRB3* (NM_139161.5) constructs were kindly provided by Prof. Masaki Nishimura (Molecular Neuroscience Research Center, Shiga University of Medical Science Molecular Neuroscience Research Center, Japan) as previously described (103). To generate N-terminal (CRB1) and C-terminal (CRB2, CRB3, and GFP) FLAG- and HA-tagged constructs, cDNA was amplified by PCR using primers carrying attb1 and attb2 recombination site overhangs. All primers were purchased from Integrated DNA Technologies IDT and amplified using Phusion High-Fidelity DNA Polymerase (Thermo Fischer Scientific). Using Gateway BP or LR Clonase II enzyme mix of the gateway cloning system (Thermo Fischer Scientific), amplicons were first recombined into pDONR201 and then into an N- or C-terminal Strep-Strep-FLAG vector as described previously (104), respectively. For HA tag, an N-terminal Strep-Strep-HA vector based on (104) or C-terminal HA vector was used. Chemically competent DH5α *E. coli* were transformed. Plasmid DNA preparation was performed using the column-based PureYieldTM Plasmid Midi Preparation Kit (A2495; Promega), and the sequence was confirmed by Sanger Sequencing (Microsynth).

### Site-directed mutagenesis

To mutate various positions in *CRB1 or CRB2*, which have been described in patients suffering from *CRB1*-linked retinal degenerations or CRB2-related syndrome, respectively, *CRB1* WT-pDONR without a start codon or CRB2 WT pDONR were amplified by PCR (5, 65). Two primers were used containing the desired mutation, and Phusion High-Fidelity DNA Polymerase (Thermo Fischer Scientific) was used for PCR amplification. After amplification, *DpnI* (New England Biolabs) digest at 37°C for 1 h was performed. Next, products were transformed with chemically competent DH5α *E. coli* and verified by Sanger Sequencing (Microsynth). Sequences of primers used for site-directed mutagenesis are shown in Table S3.

Table S3 List and sequences of primers used to introduce missense mutations in the canonical human CRB1 and CRB2 construct by site-directed mutagenesis.

### Co-immunoprecipitation

HEK293T cells were seeded in 14-cm dishes and, at a confluence of 60–80%, co-transfected with HA and FLAG constructs using home-made polyethylenimine solution as described previously (105) and incubated for 48 h at 37°C. After 48 h, cells were washed with PBS (Dulbecco’s phosphate buffered saline; Gibco) and lysed using 1 ml of lysis buffer containing 0.5% Nonidet P40 (Roche), 1% phosphatase inhibitor cocktail 2 and 3 (Sigma-Aldrich), and 2% protease inhibitor cocktail (Roche) in TBS (30 mM Tris–HCl, pH 7.4, and 150 mM NaCl). Cells were scraped, transferred into a 2-ml Eppendorf tube, and incubated for 30 min at 35 rpm at 4°C in an end-over-end shaker (neoLabLine, 7-0045). Next, samples were centrifuged at 10,000*g* for 10 min at 4°C, and the supernatant was transferred to a new tube. Protein concentration of the lysate was assessed using Bradford Assay (Bio-Rad). Part of the lysate was kept to check transfection efficiency of the input by Western blot analysis. For FLAG-IP, FLAG beads (ANTI-FLAG M2 Affinity Gel; Sigma-Aldrich) were washed once with TBS, one time with lysis buffer, and two times with washing buffer (1x TBS, 0.5% Nonidet P40 [Roche], 1% phosphatase inhibitor cocktail 2 and 3 [Sigma-Aldrich]). Equal amount of protein was loaded on 25 μl of 50% slurry FLAG beads and incubated for 60–90 min at 4°C in an end-over-end shaker. Next, bead-lysate mixtures were transferred to Receiver Columns (Macherey-Nagel) and washed three times with washing buffer, and bound proteins were eluted using the FLAG peptide (Sigma-Aldrich) in TBS following the manufacturer’s protocol. Eluates were analyzed by Western blot analysis.

### Western blot analysis

Equal amounts of protein were separated in Laemmli buffer by SDS–PAGE and transferred onto a polyvinylidene difluoride membrane following standard procedures. Membranes were blocked with 5% milk in PBS with Tween 20 (PBS-T) for 1 h at RT. Primary antibodies were incubated at overnight 4°C or 1 h at RT. The following primary antibodies were used: primary antibodies FLAG M2-Peroxidase (rabbit; Sigma-Aldrich) and anti-HA (rabbit; Cell Signaling). Secondary horseradish peroxidise–conjugated anti‐rabbit antibodies (Molecular Probes) were incubated for 1 h at RT. The ECL system (Thermo Fisher Scientific) was used to visualize proteins. Images were acquired using the Chemi Imager Fusion FX7 (Vilber) with the Image Lab software 6.0.1 (Bio-Rad). Analysis was performed with ImageJ (106).

### Porcine retina pull-down approach

HEK293T cells were seeded in a 14-cm dish. At a confluence of 60–80%, cells were transfected with 8,000 ng for DNA GFP-FLAG or CRB2-FLAG construct using home-made polyethylenimine solution and incubated for 48 h at 37°C. 48 h after transfection, cells were lysed as previously described for co-IP, and protein concentration was assessed by using Bradford Assay. FLAG beads were washed as previously described for co-IP. Per sample, 8 mg of total protein was incubated with 25 μl of 50% slurry beads for 90 min at 4°C in an end-over-end shaker. Samples were centrifuged for 1 min at 5,000*g*, the supernatant was discarded, and beads were washed twice with washing buffer. Beads were incubated with 0.01% SDS solution in TBS for 3 min at room temperature, followed by three washes with washing buffer. Next, samples were incubated with 4 mg of pig retina lysate or lysis buffer for 90 min at 4°C in an end-over-end shaker, transferred to Receiver Columns (Macherey-Nagel), and washed three times with washing buffer, and bound proteins were eluted using the FLAG peptide (Sigma-Aldrich) in TBS.

### Pig retina lysate preparation

Pig eyes were received from the slaughter house and kept at 4°C until preparation. After dissection of the eye, cold PBS was added. The neural retina without the RPE was isolated and transferred to cold lysis buffer. The neural retina of four eyes were pooled and stored at −80°C. For lysis, samples were incubated at 4°C for 30 min in an end-over-end shaker at 35 rpm and centrifuged at 10,000*g* at 4°C for 10 min, and supernatant was transferred to a new tube. For pull-down, 4 mg were used per sample.

### Mass spectrometry

Affinity purified eluates were precipitated with acetone-based protein precipitation and digested with trypsin as described previously (104). LC-MS/MS analysis was done using an UltiMate3000 RSLCnano system (Thermo Fisher Scientific) coupled to an Orbitrap Fusion Tribrid Mass Spectrometer (Thermo Fisher Scientific) by a nanospray ion source. Prepared tryptic peptide mixtures were injected automatically and loaded onto a nano-trap column (2 × 10 mm, μPACTM Trapping column, 300 nm, 100–200 Å, PharmaFluidics) with a flow rate of 10 μl/min in 0.1% trifluoroacetic acid in HPLC-grade water. After 3 min, peptides were eluted and separated on an analytical 50-cm μPACTM C18 nano column (315 μm × 50 cm, 300 nm, 100–200 Å, PharmaFluidics) by a linear gradient from 2% to 30% of buffer B (80% acetonitrile and 0.08% formic acid in HPLC-grade water) in buffer A (2% acetonitrile and 0.1% formic acid in HPLC-grade water) at a flow rate of 300 nl/min over 147 min. Remaining peptides were eluted using a step gradient from 30% to 95% buffer B in 5 min, followed by 5 min at constant 95% of buffer B. Next, the gradient was decreased rapidly in 5 min to 2% of solvent B for the final 20 min. For data-dependent analysis, full-scan MS spectra were detected on the Fusion with a resolution of 70,000 in a mass-charge range from m/z 335–1,500 with a cycle time of 3 s. The n most abundant precursor ions were selected with a quadrupole mass filter if they exceeded an intensity threshold range of 5 × 10^3^– 1 × 10^20^ and were at least doubly charged for further fragmentation using higher energy collisional dissociation. Next, mass of the fragments was analyzed in the ion trap, and the selected ions were excluded for further fragmentation the following 60 s by dynamic exclusion.

### Data analysis and statistical analysis

MS/MS data were analyzed using the MaxQuant software (version 2.0.1.0) (107, 108). Trypsin/P was selected as a digesting enzyme with a maximum of 2 missed cleavages. Cysteine carbamidomethylation or oxidation of methionine and N-terminal acetylation were selected for fixed or variable modifications, respectively. Data were analyzed using label-free quantification (no fast LFQ), and the minimum ratio count was set at 2. First search peptide tolerance was set to 20. Main search peptide tolerance was set at 4.5 ppm, and the re-quantify option was chosen. The Uniprot sus scrofa proteome database (January 2020; 49793 entries) was used, and contaminants were detected using MaxQuant contaminant search. Human CRB2 (hCRB2), CRB1 (hCRB1), CRB3 (hCRB3) and GFP sequence were added to the database. A minimum peptide number of one with minimum length of seven amino acids was required. Unique and razor peptides were used for quantification. Match between run options was selected with a match time window of 0.7 min and an alignment time window of 20 min. Statistical analysis was performed using Perseus Software (version 1.6.5.0 (109)). Six biological replicates were used for statistics, which represent individual tissue-specific IP samples processed in two independent experiments. Data were filtered for potential contaminants, peptides only identified by side or reverse sequence. In the groups, four out of six samples should have valid values. Mean values were calculated, and the stability of protein enrichment within groups was determined using the *t* test (FDR 0.05). Only proteins with a significance *P* < 0.05 and a log_2_ ratio against GFP control >2 were defined as significantly enriched/depleted. For GO enrichment analysis, the graphical tool ShinyGO 0.77 was used (60). The following settings were used: FDR cutoff 0.05, pathway size 20–250, remove redundant pathways.

## Supplementary Material

Reviewer comments

## Data Availability

The mass spectrometry proteomics data have been deposited to the ProteomeXchange Consortium via the PRIDE partner repository with the dataset identifier PXD044351 (110).
